# Chronic Hepatitis B Virus Infection: The Relation between Hepatitis B Antigen Expression, Telomere Length, Senescence, Inflammation and Fibrosis

**DOI:** 10.1371/journal.pone.0127511

**Published:** 2015-05-29

**Authors:** Phaedra M. Tachtatzis, Aileen Marshall, Aloysius Aravinthan, Suman Verma, Sue Penrhyn-Lowe, Marianna Mela, Cinzia Scarpini, Susan E. Davies, Nicholas Coleman, Graeme J. M. Alexander

**Affiliations:** 1 Department of Hepatology, St James’s University Hospital, Leeds, United Kingdom; 2 Department of Hepatology, Royal Free Hospital, London, United Kingdom; 3 Division of Gastroenterology and Hepatology, University of Cambridge, Cambridge, United Kingdom; 4 Institute of Liver Studies, King’s College Hospital, London, United Kingdom; 5 Hutchison/MRC Research Centre, University of Cambridge, Cambridge, United Kingdom; 6 Department of Pathology, University of Cambridge, Cambridge, United Kingdom; 7 Pancreatic Cancer Centre, University of Cambridge, Cambridge, United Kingdom; Singapore Institute for Clinical Sciences, SINGAPORE

## Abstract

**Background:**

Chronic Hepatitis B virus (HBV) infection can lead to the development of chronic hepatitis, cirrhosis and hepatocellular carcinoma. We hypothesized that HBV might accelerate hepatocyte ageing and investigated the effect of HBV on hepatocyte cell cycle state and biological age. We also investigated the relation between inflammation, fibrosis and cell cycle phase.

**Methods:**

Liver samples from patients with chronic HBV (n = 91), normal liver (n = 55) and regenerating liver (n = 15) were studied. Immunohistochemistry for cell cycle phase markers and HBV antigens was used to determine host cell cycle phase. Hepatocyte-specific telomere length was evaluated by quantitative fluorescent in-situ hybridization (Q-FISH) in conjunction with hepatocyte nuclear area and HBV antigen expression. The effects of induced cell cycle arrest and induced cellular senescence on HBV production were assessed *in vitro*.

**Results:**

13.7% hepatocytes in chronic HBV had entered cell cycle, but expression of markers for S, G2 and M phase was low compared with regenerating liver. Hepatocyte p21 expression was increased (10.9%) in chronic HBV and correlated with liver fibrosis. Mean telomere length was reduced in chronic HBV compared to normal. However, within HBV-affected livers, hepatocytes expressing HBV antigens had longer telomeres. Telomere length declined and hepatocyte nuclear size increased as HBV core antigen (HBcAg) expression shifted from the nucleus to cytoplasm. Nuclear co-expression of HBcAg and p21 was not observed. Cell cycle arrest induced *in vitro *was associated with increased HBV production, in contrast to  *in vitro *induction of cellular senescence, which had no effect.

**Conclusion:**

Chronic HBV infection was associated with hepatocyte G1 cell cycle arrest and accelerated hepatocyte ageing, implying that HBV induced cellular senescence. However, HBV replication was confined to biologically younger hepatocytes. Changes in the cellular location of HBcAg may be related to the onset of cellular senescence.

## Introduction

Both the age of onset and the severity of liver injury in chronic hepatitis B virus (HBV) infection vary widely, from asymptomatic lifelong carriage to chronic hepatitis, cirrhosis and hepatocellular carcinoma (HCC) [1]. There is a strong, unexplained correlation between increasing age and the risk of cirrhosis or HCC [2].

The number of hepatocytes that express HBV antigens and their cellular distribution vary with disease duration and disease progression [3, 4]. In the early ‘immune tolerant’ phase there is strong nuclear HBcAg expression, with high titre HBV DNA, HBeAg and HBsAg in serum. In the later ‘reactive’ phase, which accompanies HBeAg/anti-HBe seroconversion, serum levels of HBV DNA and HBsAg fall, with a shift to cytoplasmic HBcAg expression [3]. Later still, serum HBsAg and HBV DNA fall to low or undetectable levels and hepatocyte HBcAg is detected rarely [4].

The marked fall in HBsAg and viral load with time and increased age contrasts with other chronic viral infections such as HCV or HIV and remains unexplained. Such changes have been attributed previously to enhanced host immune responses to HBV, but there is little evidence that these are augmented with increased duration of infection or increasing age.

Hepatocyte senescence has been described in chronic liver disease and attributed to increased hepatocyte turnover, often termed ‘replicative senescence’ [5], yet mitotic figures are rare in chronic HBV infection. Hepatocyte cell cycle arrest, an alternative pathway leading to premature hepatocyte senescence, is a feature of chronic HCV infection [6], non alcohol-related fatty liver disease [7] and alcohol-related liver disease [8]. In these conditions increased hepatocyte expression of p21, a universal cell cycle inhibitor, plays a key role in induction and maintenance of cellular senescence where hepatocyte p21 expression correlates with fibrosis stage and an adverse clinical outcome [6–8].

The purpose of the study was to determine if accelerated ageing and senescence were present in liver tissue from patients with chronic HBV infection and to establish the relation between HBV antigen expression and ageing, senescence, inflammation and fibrosis.

## Materials and Methods

### Ethics

Archived human liver tissue was obtained from the Tissue Bank at Addenbrooke’s Hospital, Cambridge (supported by the BRC) and St James’s University Hospital, Leeds. Written consent for the use of liver tissue for research purposes and for archiving was obtained at the time of consenting for the procedure. Both Cambridge and Norfolk & Norwich Research Ethics Committees approved the study.

### Patient sections

Chronic HBV infection was defined as the presence of HBsAg in serum for over 12 months and patients were selected such that a comprehensive screen for other acute or chronic liver disease was negative. None of the patients received antiviral therapy. Overall, sections were available for study from 91 patients representing the full spectrum of inflammation and fibrosis associated with chronic HBV infection. Liver sections were selected from this cohort to address particular questions according to patient classification. Patients were classified according to replication status: HBeAg-positive/anti-HBe negative and positive for HBV DNA in serum, HBeAg negative/anti-HBe positive and positive for HBV DNA in serum and anti-HBe positive but negative for HBV DNA in serum. Further classification was based on the hepatic activity index (HAI) and fibrosis stage. (S1 Table). Where comparisons are made these are with age and gender matched groups.

Every liver biopsy was performed as part of routine clinical assessment and reviewed by an experienced histopathologist (SED). Biopsies were scored according to modified Ishak criteria [9] after assessing specimen adequacy: interface hepatitis 0–4; lobular inflammation 0–4; portal inflammation 0–4; fibrosis was scored 0 (absent)- 6 (cirrhosis); steatosis 0–4; the histological activity index (HAI) was derived from these scores. HBsAg and HBcAg were scored qualitatively.

### Control liver sections for cell cycle phase experiments

Selected liver sections from archived formalin-fixed paraffin-embedded liver needle biopsy specimens served as control tissue as described previously [6]. Ten patients undergoing liver resection for distant metastases (who had not received chemotherapy) with normal liver histology served as *negative* controls. Fifteen patients undergoing liver biopsy in the early post operative period after liver transplantation with histological evidence only of hepatic regeneration were selected from a larger series because liver function tests returned to the normal range promptly and these served as *positive* controls representing a discrete episode of liver inflammation with resolution as described previously [6].

### Control liver sections for Q-FISH experiments

Liver sections from 55 archived formalin-fixed paraffin-embedded liver needle biopsy specimens from age-matched donor livers at the time of liver transplantation (time-zero biopsies), served as controls, as described in detail previously [10]. These sections of adequate size were chosen to reflect normal liver on the basis of satisfying all of the following criteria: no history of liver disease or senescence-related disease in the donor; a short medical illness preceding donor death; no or minimal reperfusion injury or steatosis at histological review; excellent graft function 1-year after transplantation.

### Virology

Serum HBsAg was sought using ADVIA Centaur immunoassay (Siemens, Surrey, UK), confirmed with polyclonal HBsAg (Vidas); HBeAg and anti-HBe with the Vidas platform (Biomerieux, Hampshire UK). Serum HBV DNA was quantified at the HPA, Addenbrooke’s Hospital, Cambridge, by LightCycler assay. HBsAg titres were measured with the Elecsys HBsAg II Kit (Id. No. 04687787).

### Immunohistochemistry, immunofluorescence and Q-FISH

Immunohistochemistry for cell cycle markers and immunofluorescence for hepatocytes was performed as described previously [6, 10]. The cell cycle markers included Mcm-2, cyclin D1, cyclin A, cyclin B1 and PH3, which represent different phases of the cell cycle. [MCM-2: cell cycle entry; cyclin D1: G1 phase; cyclin A: S phase; cyclin B1: G2 phase; PH3: transition from G2 through mitosis]. In addition immunohistochemistry was used to detect p21, a cdk inhibitor, which blocks cell cycle progression at G1.

Immunofluorescence for HBV antigens was carried out in both cell cycle phase and Q-FISH experiments using similar methods with a series of murine monoclonal antibodies to HBV antigens. All antibodies to identify cell cycle phase markers, hepatocytes, HBsAg, HBcAg and HBeAg are listed in S2 Table.

Telomere-specific probes were hybridised to liver sections and images captured using the Olympus ScanR High Content Screening platform. Telomere number, telomere intensity (a measure of telomere length) and nuclear area of hepatocytes were determined using Q-FISH as described previously [10]. Nuclear area was calculated as described previously [10]. Hepatocytes were identified using antibody to Hepar-1 (S2 Table).

### Assessment of HBV antigen production in relation to cell cycle arrest and cellular senescence in hepG2 and hepG2.2.15 cell lines

HepG2 and hepG2.2.15 cell lines were used to assess the effect of cell cycle arrest and cellular senescence on HBV production. HepG2.2.15 cells are transfected stably with sub-genomic HBV; thus HBV production is a result of transcription of the transgene. Aphidicolin was used to induce G1/S arrest and camptothecin to induce G2/M arrest. Brief exposure to 1mM hydrogen peroxide was used to induce cellular senescence; the cell lines are immortal so do not undergo replicative senescence on serial passages. Cell cycle distribution was assessed by flow cytometric analysis of DNA content and 5-bromo-2'-deoxyuridine (BrdU) incorporation. Cellular senescence was assessed by morphology, senescence-associated β-galactosidase staining and BrdU incorporation. HBsAg production by HepG2.2.15 cells was assessed in supernatant using a commercial enzyme immunoassay (AxSYM HBsAg V2, Abbott, Germany). Supernatant HBV DNA was measured with an in-house RT-PCR assay. HBcAg was assayed by immunofluorescence. Since variation in supernatant HBsAg and HBV DNA could be explained by variation in final cell numbers HBsAg and HBV DNA were corrected for cell number per well using lactate dehydrogenase activity.

### Statistics

Statistical analysis was performed on GRAPH PAD Prism5 software (Graph Pad, San Diego, USA) and SPSS (version 18) using the linear regression test as data demonstrated a normal distribution. A p-value of 0.05 or less was considered significant. Two-tailed unpaired t-tests were used to define differences between means of normally distributed data of equal variance. A Mann-Whitney test was used for data that were not distributed normally. Wilcoxon matched-pairs signed rank test was used to compare different populations of cells within the same tissue. One-way ANOVA test was used for comparison between multiple groups and correlations between variables were calculated using logistic regression analysis and a linear regression model.

## Results

### Cell cycle phase markers (n = 39)

**Fig 1:**
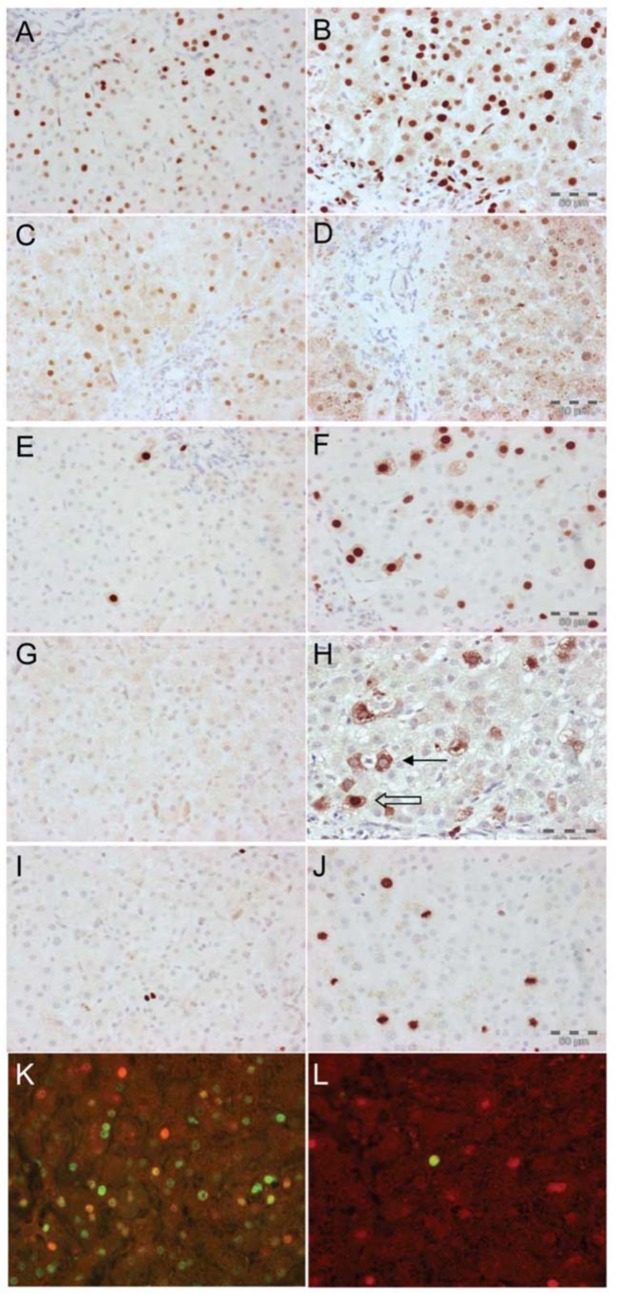
Hepatocytes are arrested in G1 in chronic HBV infection. Immunoperoxidase staining using cell cycle phase-specific antibodies in a representative case of chronic HBV infection A—E. Immunofluorescence showing the relation between HBcAg expression and Mcm-2 (F) and cyclin A (G). A: Mcm-2 identifies cells that have entered the cycle. B: Cyclin D1, expression is maximal during G1. C: Cyclin A is expressed maximally during S phase. D: Cytoplasmic expression of Cyclin B1 during G2, nuclear and cytoplasmic expression. E: Phosphorylated histone 3 protein expressed in mitosis. F: Immunofluorescence for Mcm-2 (green) and HBcAg (red), showing frequent co-localisation (yellow). G: Immunofluorescence for cyclin A (green) and HBcAg (red). Co-localisation was never seen.

Representative experiments for cell cycle phase markers in patients with chronic HBV infection are shown in Fig 1. Hepatocyte Mcm-2 expression was higher in regeneration compared with chronic HBV infection (26% versus 13.7% respectively, p = 0.02). Hepatocyte cyclin D1 expression was similar in both (53% and 43% of Mcm-2 positive hepatocytes respectively p = 0.26). In contrast hepatocyte expression of Cyclin A, Cyclin B1 and PH3, were all higher in regeneration compared to chronic HBV infection {cyclin A 16% vs. 3% (range 2–37% vs. 0–67%), p < 0.0001, cyclin B1 2% vs. 0% (range 0.3–10% vs. 0–28%), p < 0.0001, and PH3 4% vs. 0.7% (range 0.3–10% vs. 0–55%), p = 0.000, see also S1 Fig). In those with chronic HBV there were strong associations between hepatocyte Mcm-2 expression and both increased fibrosis stage (p = 0.02) and increased inflammation (p = 0.007).

Median hepatocyte p21 expression was increased in regeneration and chronic HBV infection (9.5% and 10.9% respectively). Median hepatocyte p21 expression was associated with increased fibrosis stage in chronic HBV infection (p = 0.006) and increased HAI (p < 0.0001).

### HBV antigen expression and cell cycle entry (n = 8)

Sections from 8 patients with chronic HBV positive for HBV DNA in serum were double-labeled for Mcm-2 and HBcAg. The median number of HBcAg positive hepatocytes also positive for Mcm-2 was 55% (range 29–93%, Fig 1).

### HBV antigen expression and cell cycle progression (n = 5)

Sections from patients with chronic HBV positive for HBV DNA in serum were double-labeled for HBcAg and cyclin A (Fig 1). In each case the whole biopsy was examined for hepatocyte co-expression of cyclin A (denoting an hepatocyte in S phase) with HBcAg, but this was never seen.

### HBcAg and p21 co-expression (n = 13)

Thirteen liver sections were further selected from patients with HBV replication, as well as liver injury, in order to ensure inclusion of hepatocytes that were known to express HBcAg and p21. Hepatocyte co-expression of HBcAg and p21 was not observed.

Thus, in chronic HBV infection, very low hepatocyte expression of S-phase markers and markers beyond S phase in the cell cycle was consistent with G1 arrest.

Expression of the cell cycle entry marker Mcm-2 was associated with increased inflammation, increased fibrosis and viral replication.

p21 which mediates cell cycle arrest was associated with increased inflammation and increased fibrosis.

Viral replication was limited to those hepatocytes that had entered the cell cycle, but was never detected in cells expressing cell cycle arrest.

### Effects of cell cycle arrest and induced cellular senescence on HBV production in HepG2.2.15 cell line

*In vitro* induced cell cycle arrest caused increased levels of supernatant HBsAg and HBV DNA. In addition increased HBcAg expression was noted in cell-cycle arrested hepatocytes. In contrast no effect on HBsAg or HBV DNA production was observed in senescent cells, with only a modest increase in cytoplasmic HBcAg staining.

### Q-FISH for telomeres (n = 39)

**Fig 2:**
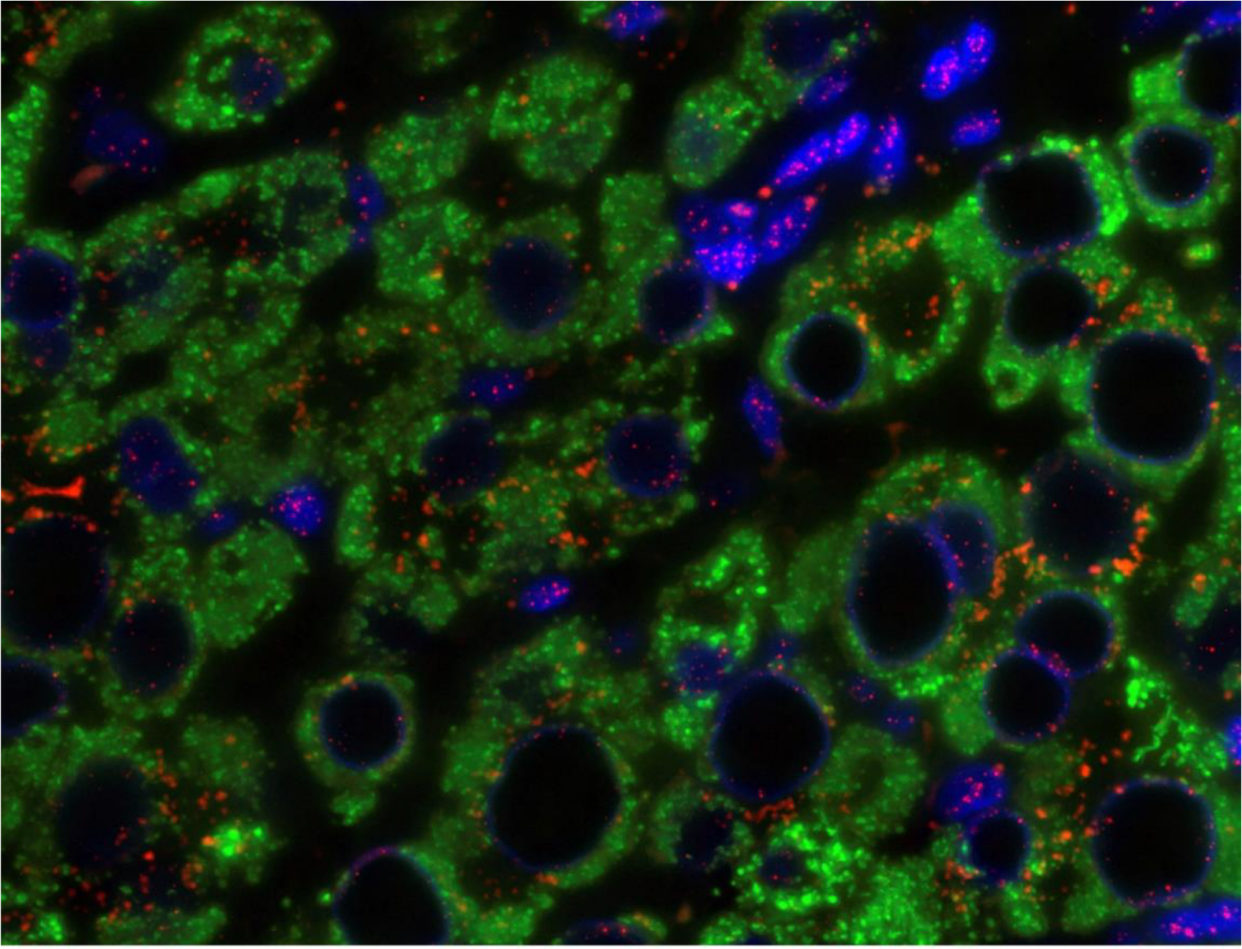
Q-FISH of liver tissue in chronic HBV infection. Hepatocytes with cytoplasm stained green with antibody to Hepar-1, nuclei stained blue with DAPI and telomeres shown in pink. Note vacuolated nuclei with peripheral telomeres as seen in senescence (13).

**Fig 3:**
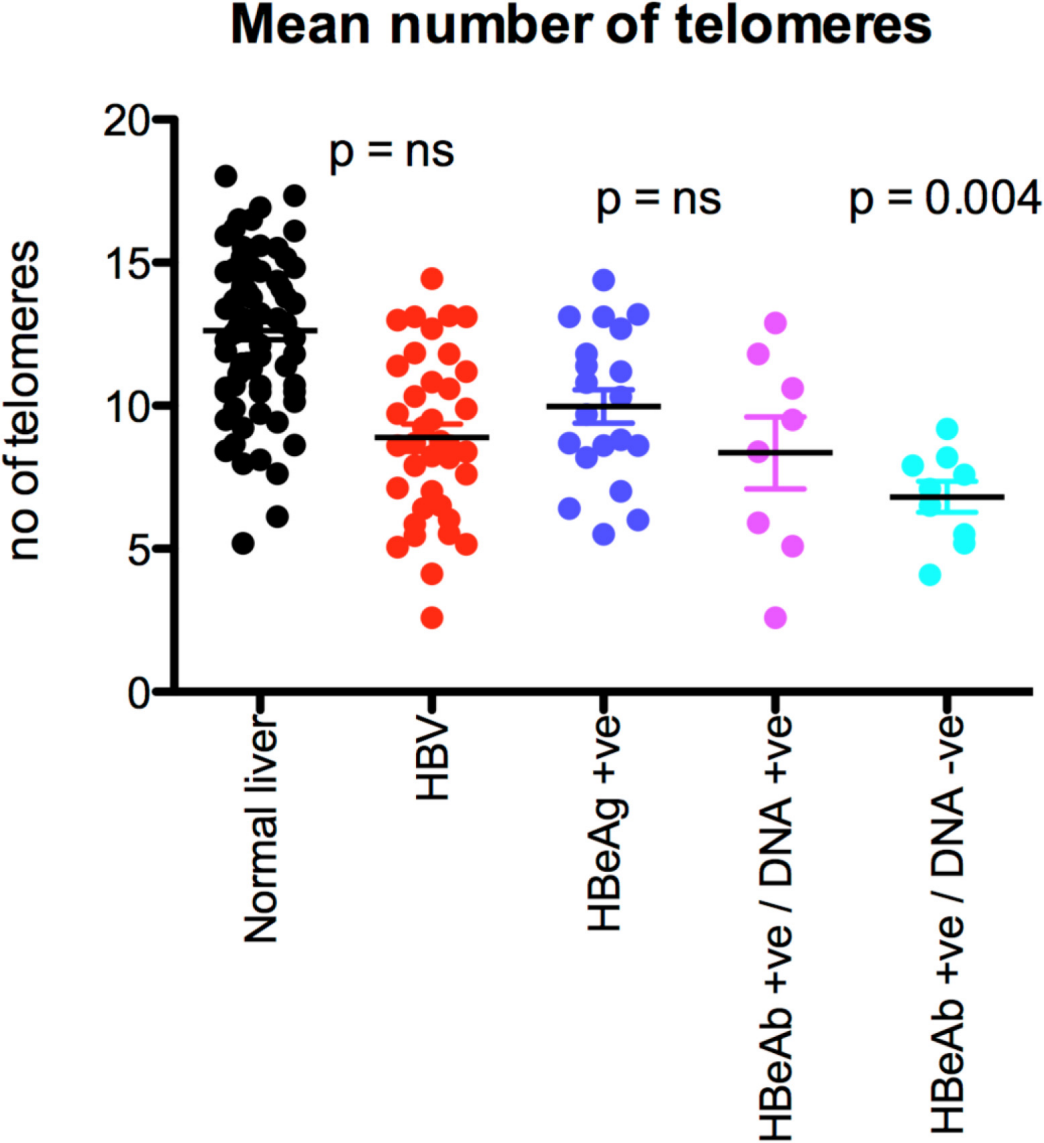
The mean number of telomeres in normal and HBV-infected liver tissue according to viral load.

Fig 2 is a representative example of hepatocyte staining in chronic HBV. Comparison was made with 55 age and sex matched sections control. Overall, the mean number of telomeres detected per hepatocyte was similar in chronic HBV infection and controls (Table 1). However, the mean number of telomeres detected per hepatocyte fell in parallel with the viral load (p = 0.04, Fig 3).

**Table 1:** Q-FISH analysis of hepatocytes.

	Chronic HBV infection		Controls	
	Mean	Standard deviation	Mean	Standard deviation
**Number of telomeres per hepatocyte**	**8.9**	**2.9**	**12.8**	**2.6**
**Mean of mean telomere length (Cy5 intensity)**	166 *	**119**	**465**	**159**
**Mean of maximum telomere lengths**	253 *	**183**	**721**	**229**
**Telomere area**	8.9 *	**1.3**	**10.2**	**1.5**
**Nuclear area in pixels (μm** ^**2**^ **)**	**4195 (48.5)**	**524**	**4205 (48.6)**	**496**

* Values for patients with HBV differed significantly from controls (all p < 0.0001).

Mean telomere length was shorter in patients with HBV than in controls (166 (SD 119) and 465 (SD 159) respectively, p < 0.0001). The mean of maximum telomere length was also shorter in patients with HBV than in controls (253 (SD 183) and 721 (SD 229) respectively, p < 0.0001). Mean telomere area was reduced in patients with HBV than in controls (8.9 (1.3) and 10.2 (SD 1.5) respectively, p < 0.0001, Table 1).

### Hepatocyte nuclear area (n = 39)

**Fig 4:**
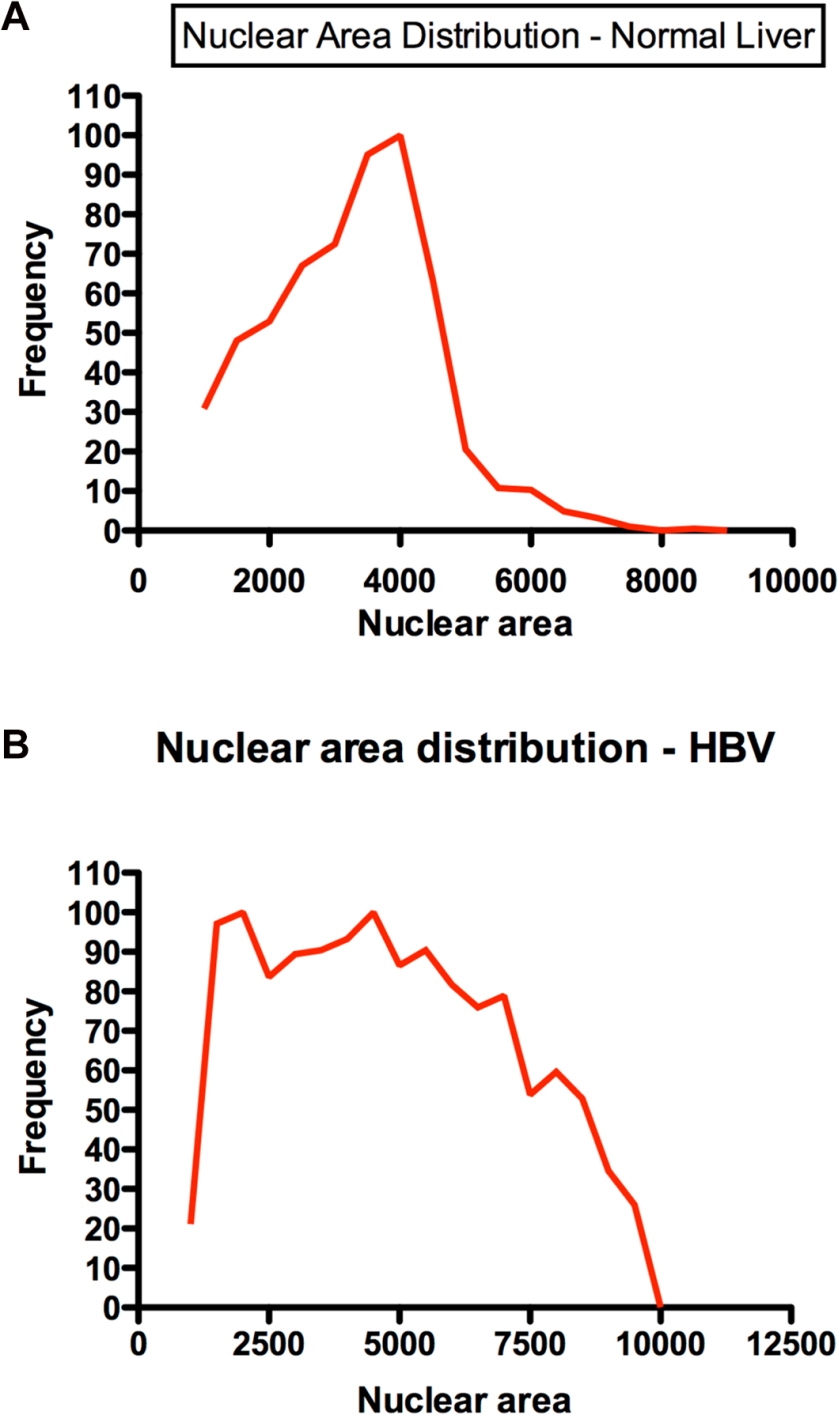
(a) Hepatocyte nuclear area in normal liver. A representative example of nuclear area distribution in normal liver. (b) Hepatocyte nuclear area in HBV infected liver. A representative example of nuclear area distribution HBV-infected liver.

Mean hepatocyte nuclear area was similar in patients with HBV and controls (4195 or 48.5 μm^2^ (SD 524) and 4205 or 48.6 μm^2^ (SD 496) respectively, Table 1). However, this finding masked considerable polymorphism in hepatocyte nuclear area in patients with chronic HBV infection when compared with controls (Fig 4A and 4B); in patients with chronic HBV infection, the proportion of hepatocytes with nuclear area less than 2 standard deviations below the mean (derived from normal liver) ranged from 16.5% to 59.9% and the proportion of hepatocytes with a nuclear area that exceeded 2 standard deviations above the mean ranged from 3.3% to 48.8%. The proportion of hepatocytes with large nuclei (more than 2 standard deviations above the mean) correlated positively with fibrosis stage (p <0.0001), but there was no correlation between fibrosis stage and the proportion of hepatocytes with smaller nuclei.

### HBsAg positive hepatocyte telomeres (n = 9)

**Fig 5:**
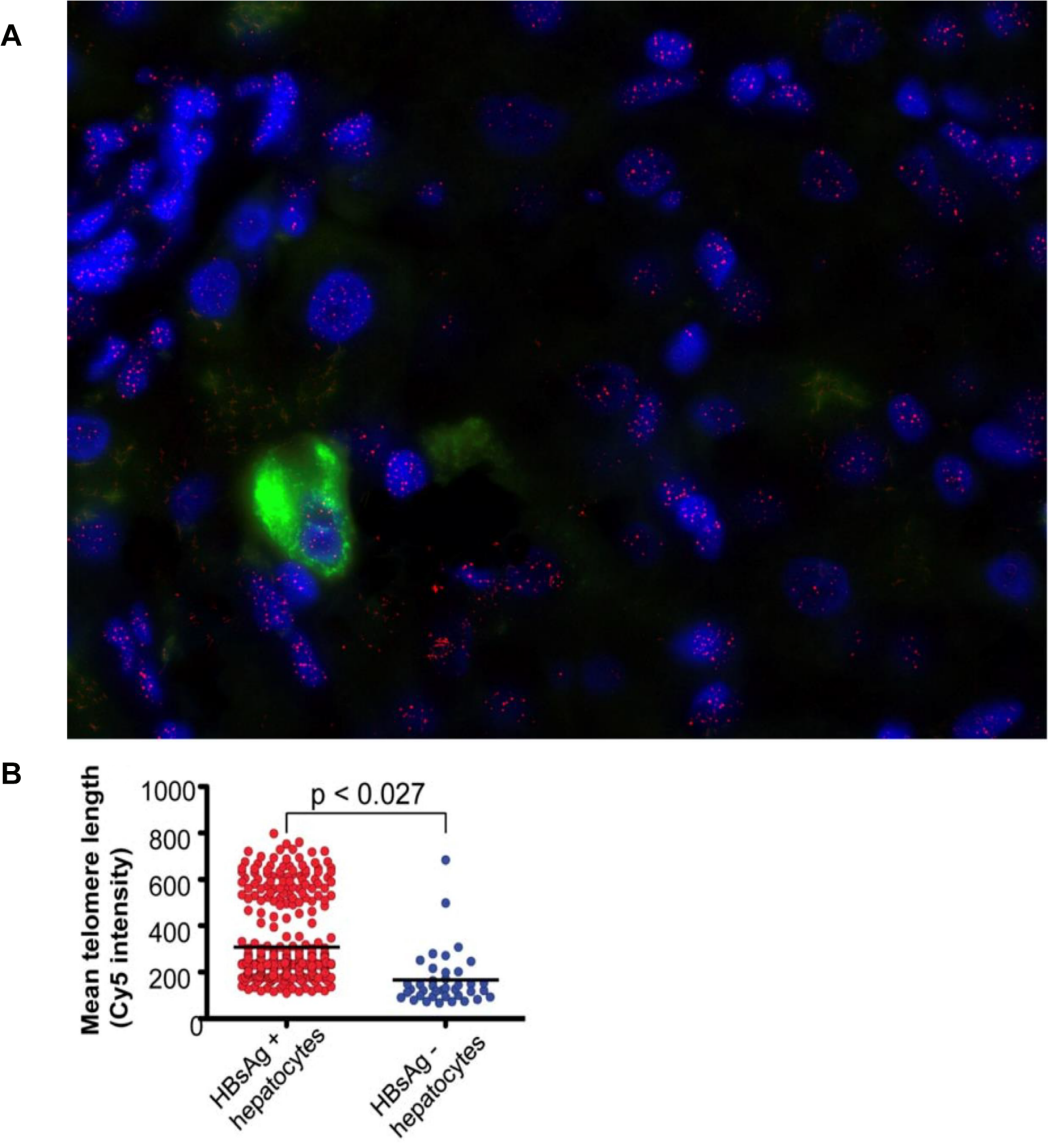
(a) A representative example of a liver stained for HBsAg in chronic HBV infection. HBsAg is detected in cytoplasm as green. Nuclei are blue(DAPI) and telomeres stain pink. (b) Mean telomere length in HBsAg+ hepatocytes versus ‘all hepatocytes’.

Liver sections were chosen such that they contained sufficient hepatocytes expressing HBsAg. Hepatocytes that expressed cytoplasmic HBsAg detected by immunohistochemistry, which could be clearly identified as distinct from neighbouring hepatocytes, were analysed separately (Fig 5A) and the results were compared with *all* hepatocytes detected with anti-Hepar-1 within the same section.

In each individual section the number of telomeres detected and the telomere length was always higher in HBsAg positive hepatocytes than in ‘all hepatocytes’. Overall, the mean number of telomeres detected in HBsAg positive hepatocytes and ‘all hepatocytes’ was (14.4 (SD 3.6) and 8.2 (SD 3.3) respectively, p = 0.004). Overall in the 9 sections, the mean telomere lengths in HBsAg positive hepatocytes and ‘all hepatocytes’ were (219 (SD 40) and 136 (SD 49) respectively, p = 0.027)(Fig 5B).

Mean nuclear area was similar in HBsAg positive hepatocytes when compared to ‘all hepatocytes’ (4420 and 3866 μm^2^ respectively, p = 0.074).

### HBcAg positive hepatocyte telomeres (n = 10)

**Fig 6:**
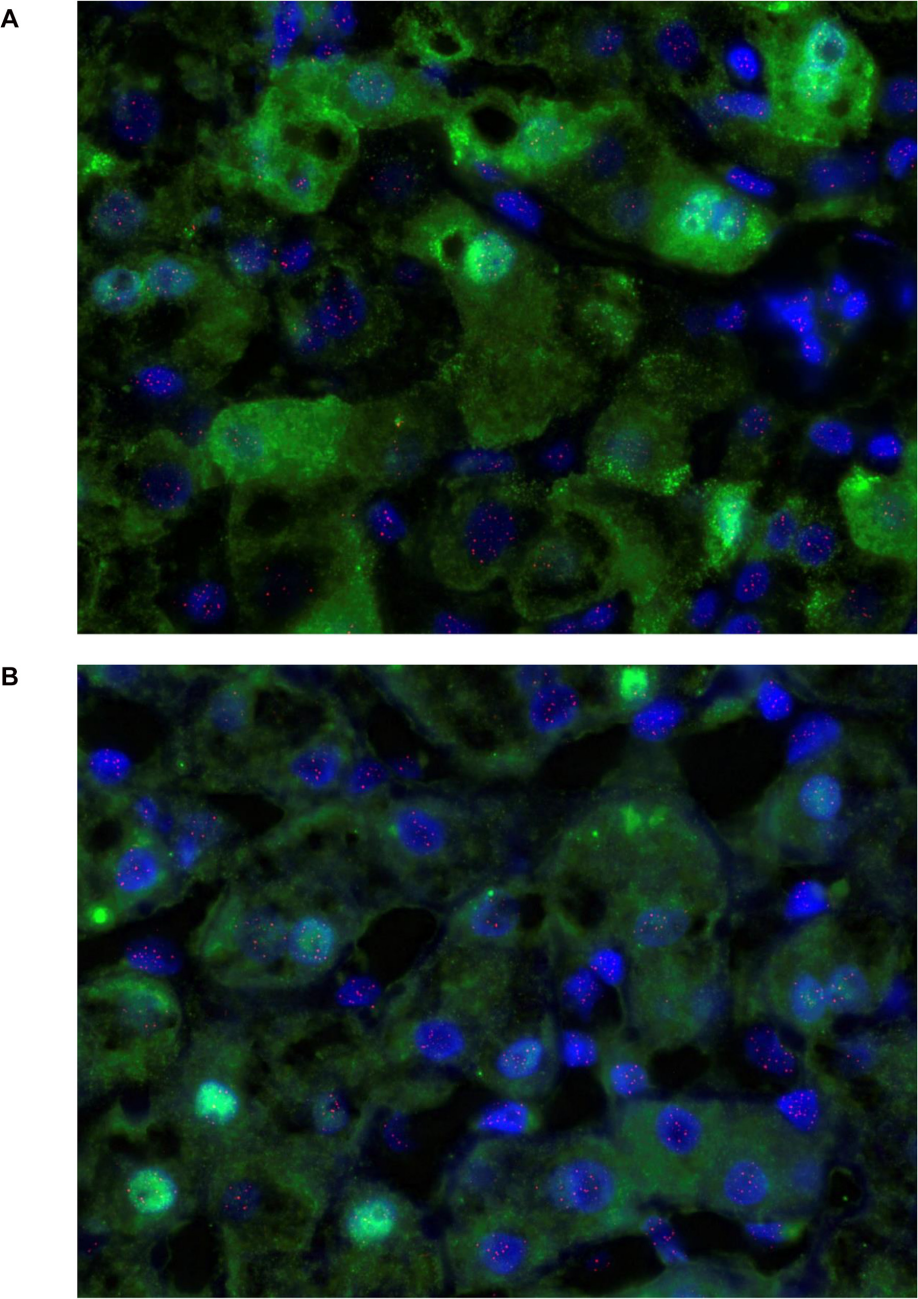
(a) Nuclear and cytoplasmic HBcAg staining detected by Q-FISH in liver from a representative patient with chronic HBV infection. HBcAg stains bright green, nuclei stain blue with DAPI and telomeres stain pink. (b) Nuclear HBcAg staining detected by Q-FISH in liver from a representative patient with chronic HBV infection. HBcAg stains bright green, nuclei stain blue with DAPI and telomeres stain pink.

Sections from patients with chronic HBV infection were selected specifically for those with detectable HBV DNA in serum. Comparison was made between hepatocytes that expressed HBcAg, detected with immunohistochemistry and ‘all hepatocytes’ in the same section detected with anti-Hepar-1 (Fig 6A and 6B). Further analysis was also carried out according to the intracellular location of HBcAg: nuclear alone, cytoplasmic alone or both nuclear and cytoplasmic.

**Fig 7:**
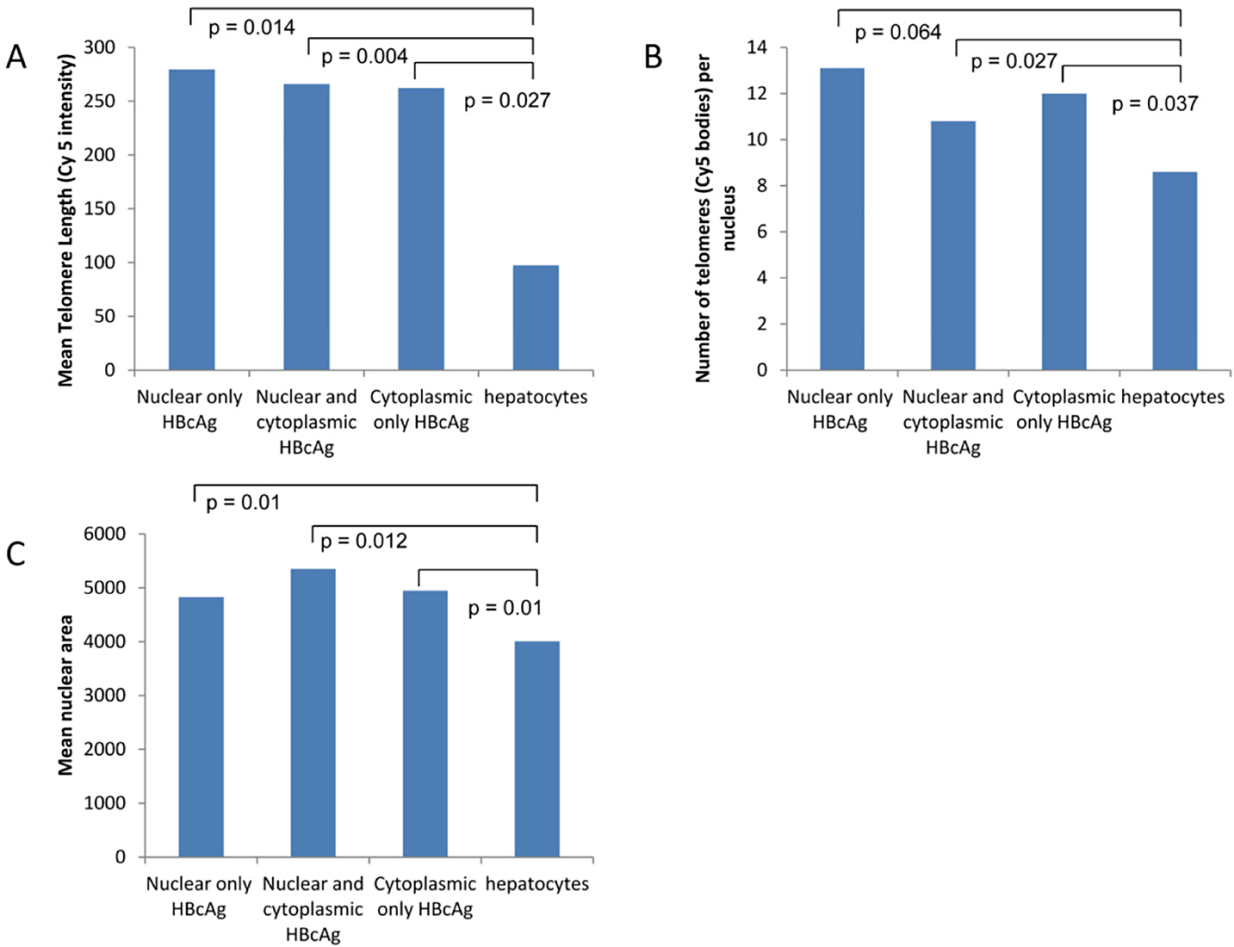
Telomere length, telomere number and nuclear area according to the subcellular location of HBcAg.

In each section mean telomere length in HBcAg positive hepatocytes always exceeded that in ‘all hepatocytes’ (Fig 7A). Overall, telomere length in hepatocytes with nuclear HBcAg expression alone was greater than in ‘all hepatocytes’ (p = 0.01, Fig 7A). Telomeres were longer with nuclear HBcAg expression alone compared to hepatocytes in which HBcAg was expressed in both nucleus and cytoplasm (p = 0.004, Fig 7A) while hepatocytes with cytoplasmic HBcAg alone had even shorter telomeres (p = 0.027, Fig 7A).

In each section the number of telomeres was always higher in HBcAg positive hepatocytes compared to ‘all hepatocytes’. There were always more telomeres in hepatocytes in which HBcAg was expressed either in the nucleus alone or in the cytoplasm alone or in both combined nucleus and cytoplasm when compared to ‘all hepatocytes’ (p = 0.064, p = 0.027, p = 0.037, Fig 7B).

Mean nuclear area in hepatocytes with nuclear HBcAg expression alone was similar to ‘all hepatocytes’. However, nuclear area was greater when HBcAg was detected in cytoplasm alone or both nuclear and cytoplasmic expression (Fig 7C, p = 0.01).

### HBeAg positive hepatocyte telomere (n = 6)

**Fig 8:**
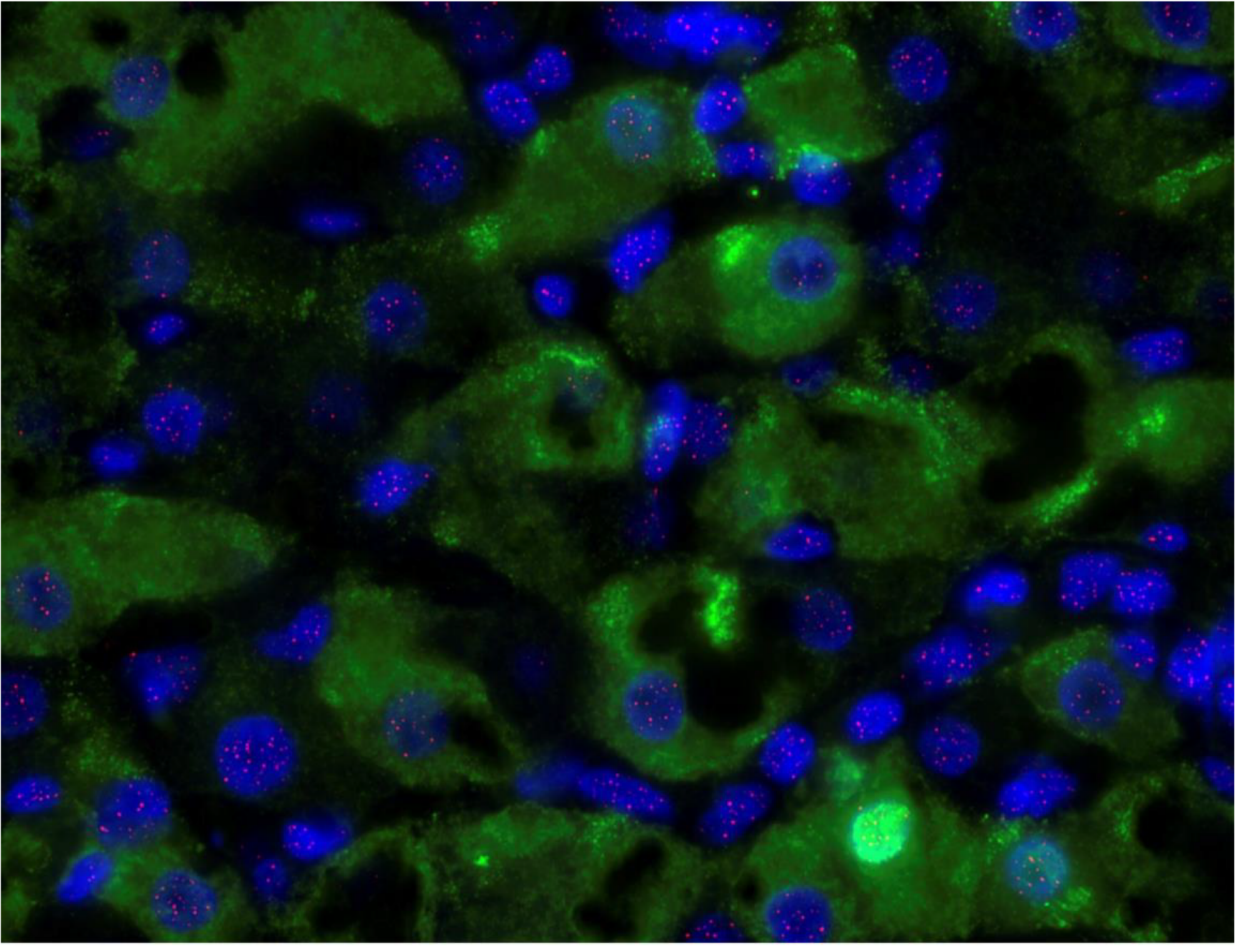
Nuclear HBeAg staining in a representative patient with chronic HBV infection. HBeAg stains bright green, nuclei stain blue with DAPI and telomeres stain pink.

Q-FISH was performed in further sections from patients with chronic HBV infection (Fig 8), selected for the presence of HBeAg in serum. Comparison was made between hepatocytes in the same section that expressed HBeAg detected by immunohistochemistry and ‘all hepatocytes’ detected with anti-Hepar-1.

Telomere length in each section was always greater in HBeAg positive hepatocytes than ‘all hepatocytes’. However, in this small series (limited by tissue availability) mean telomere length in HBeAg positive hepatocytes was similar to that in ‘all hepatocytes’ (p = 0.13), while the number of telomeres was greater in HBeAg positive hepatocytes than ‘all hepatocytes’ (p = 0.004, data not shown).

### Linear regression analysis (n = 39)

This was used to investigate associations between clinical, demographic or histology data including age, sex, albumin, bilirubin, ALT, PT, HBeAg/anti-HBe status, HBV-DNA status, HBsAg titre, the presence of interface hepatitis, portal tract inflammation, lobular inflammation, steatosis, fibrosis stage, hepatocyte expression of HBsAg/HBcAg with data derived from Q-FISH including the number of telomeres, mean telomere length, maximum telomere length and nuclear area. Only significant associations are reported.

In chronic HBV infection serum albumin and age were associated with the number of telomeres detected (p < 0.05); the number of telomeres detected fell with increasing age after adjusting for albumin. Unexpectedly, higher levels of serum albumin were associated with reduced telomere number after adjusting for age. Increased steatosis was associated with shorter telomeres (p < 0.01) and reduced maximal telomere intensity (p < 0.02) but did not correlate with fibrosis. Portal tract inflammation and fibrosis stage were related to nuclear area (p < 0.05). Increased portal tract inflammation was related to increased nuclear area after adjusting for staging (p = 0.029). Increased fibrosis stage was related to increased nuclear area after adjusting for portal tract inflammation (p = 0.0003). Higher serum albumin and decreasing age were associated with smaller hepatocyte nuclei (p < 0.05) after adjusting for age and albumin respectively.

## Discussion

This study presents key findings, which are consistent with the clinical pattern of HBV replication over many years and link HBV replication, antigen expression, inflammation and fibrosis to biological ageing of the hepatocyte.

Hepatocytes in patients with chronic HBV infection had evidence of cell cycle arrest in G1; HBV replication was confined to cells that had entered the cell cycle; cells unable to complete the cell cycle (p21 positive) were never positive for markers of HBV replication; the proportion of hepatocytes that expressed either Mcm-2 or p21 correlated with hepatic inflammation and fibrosis stage.

Induction of cell cycle arrest in an HBV-transfected cell line led to increased HBV production (S2 Fig), consistent with previous findings [11]. Further, induction of cell cycle arrest in an HBV-transfected cell line was found to correlate with a shift in expression of HBcAg to the cytoplasm. These are *in vitro* findings in a malignant cell line and support the clinical relation between HBV replication and cell cycle arrest and senescence. Cell cycle arrest in G1 or G2 is known to provide an environment conducive to enhanced replication for many viruses; manipulation of host cell cycle state or telomerase expression to enhance viral replication has been described for HCV, CMV, HIV, EBV, HHV-8, HPV and HTLV-1 [6,12].

Telomeres are repetitive sequences that protect the ends of linear chromosomes, but small telomere sequences are lost during each round of cell division; thus telomere length is a crude measure of cell turnover and therefore of ‘biological age’. This may at a critical point trigger the DNA damage response. Terminally differentiated cells, such as hepatocytes, replicate infrequently in health and hepatocyte telomeres do not shorten with age in healthy liver [10].

Fewer telomeres were detected in patients with chronic HBV infection compared to controls (presumably a reflection of telomeres below the level of detection) and when telomeres were detected they were shorter than in matched controls. However, within each HBV infected liver, hepatocytes expressing HBV antigens had a greater number of telomeres and longer telomeres than HBV antigen negative hepatocytes. This finding was more marked in those hepatocytes with nuclear HBcAg alone; hepatocyte telomere length and number fell as the location of HBcAg moved from nucleus to the cytoplasm. Thus the greatest number of telomeres and the longest telomeres were found in those with markers of a higher viral load i.e. the presence of HBeAg and HBV DNA in serum, as well as nuclear HBcAg. Increased nuclear area is a recognised marker of senescence [13]. In this series hepatocyte nuclear area correlated closely with both inflammation and fibrosis stage and was greater in hepatocytes with cytoplasmic HBcAg as opposed to nuclear HBcAg expression.

Widespread hepatocyte telomere shortening is consistent with accelerated ageing, which has been described in chronic HBV [14] but also in liver cirrhosis due to other aetiologies [5]. Until recently, telomere shortening observed in liver cirrhosis was thought to be due to increased cell turnover resulting in replicative senescence. However, accumulating evidence data shows that cirrhosis is more often associated with cell cycle arrest. Prolonged cell cycle arrest can lead to cellular senescence directly. This has been shown in non-alcohol-related liver disease [7], alcohol-related liver disease [8] and chronic HCV infection [6]. Our study lends further support to a similar phenomenon occurring in chronic HBV infection.

HBV antigens were detected in hepatocytes that had longer telomeres than background liver, albeit shorter than in health. This suggests either HBV entry or HBV replication is less efficient in ‘older hepatocytes’ and is compatible with the natural clinical history of HBV infection with substantial falls in both serum HBV DNA and HBsAg titre with each decade. HBeAg loss usually occurs, although at a variable age between individuals [15, 16] and is accelerated by antiviral therapy. Although HBsAg titres fall with age, HBsAg loss is less frequent with or without antiviral therapy and if it occurs, then it is many years later than HBeAg loss.

Since host immune responses, indicated by impaired T-cell responses to HBV antigens and mitogens tend to weaken and narrow with time [17–20] it seems less likely that a stronger host immune responses can account for this gradual decline in HBV replication. A virus-driven change in HBV antigen expression in relation to increasing age and duration of infection seems more logical.

The change in cellular HBV antigen localisation with disease progression, first reported many years ago [3], is also consistent with the age driven changes in HBV expression described here. Nuclear HBcAg expression, characteristic of the early “immune tolerant phase” was associated with the longest telomeres, while cytoplasmic HBcAg expression (seen with more advanced disease) was associated with shorter telomeres relative to those with nuclear HBcAg. Furthermore, the total number of hepatocyte telomeres fell with progression from the early stages of chronic HBV infection (HBeAg positive) to late stage (anti-HBe positive with undetectable HBV DNA). These results indicate that temporal changes in the cellular location of HBV antigens correlate with increasing biological age of hepatocytes.

Furthermore, the nuclear size of hepatocytes increased as HBcAg expression shifted to the cytoplasm and there co-localisation between p21, a marker of senescence and HBcAg expression was never seen. These results suggest that the increasing biological age of hepatocytes and possibly the onset of cellular senescence are related to changes in the location of the HBV antigens. The mechanisms underlying the location of HBcAg in more advanced disease in man or with cellular senescence induced *in vitro* point to altered intracellular transport.

It is also possible that the intense inflammatory process that occurs during HBeAg/anti-HBe seroconversion and which often leads to severe fibrosis may be due to ‘ageing hepatocytes’ and onset of cellular senescence. For many decades it was believed that it was due to enhanced immune responses after decades of HBV infection. Hepatocyte senescence is pro-inflammatory and in this series both p21 and nuclear area, which are close correlates of senescence, were related to inflammation as well as fibrosis. This may be mediated by the senescence-associated secretory phenotype (SASP), involving secretion of numerous cytokines, growth factors and proteases by senescent cells, which is well described and linked to inflammation and fibrosis in other diseases [21].

Hepatocyte cell cycle arrest and senescence appear to be common to several chronic liver diseases but there are some differences. In NAFLD and ALD, the frequency of hepatocyte p21 expression was much higher [7, 8] than hepatocyte Mcm-2 expression; in viral hepatitis hepatocyte p21 and Mcm-2 expression were similar [6]. The viruses may induce cell cycle arrest, following which Mcm-2 is lost eventually [22] and hepatocytes then become senescent. In contrast, both non-alcohol related fatty liver and alcohol related liver diseases are characterised by oxidative stress, which might induce hepatocyte senescence directly. Steatosis may be a feature of chronic HBV infection; in this series hepatic steatosis correlated inversely with telomere length, despite inclusion of HBV patients with a normal BMI and without diabetes mellitus or hyperlipidaemia. Thus steatosis might exacerbate accelerated hepatocyte ageing and senescence in patients with HBV infection.

In conclusion, we have shown that chronic HBV infection induces G1 arrest mediated by p21. Cell cycle entry and cell cycle arrest correlated positively with inflammation and fibrosis. HBV replication was confined to younger hepatocytes in cell cycle arrest amongst a population of more aged hepatocytes. The shift of the HBcAg location from the nucleus to the cytoplasm related to the onset of cellular senescence.

## Supporting Information

S1 Fig Hepatocyte Mcm-2 and cell cycle phase markers comparing liver from 39 patients with chronic HBV and 15 with liver regeneration. (A) % Mcm-2 positive hepatocytes in HBV versus liver regeneration, p = 0.02. (B)–(E) show results expressed as % of Mcm-2 positive hepatocytes for each case. (B) Cyclin D1, p = 0.25 (C) Cyclin A, p < 0.0001 (D) Cyclin B1, p < 0.0001 (E) PH3, p = 0. 0004. The black bar in the middle of the box represents the median, the box stretches between the lower and upper quartiles and the whiskers extend to the range of the data or 1.5 times the box length whichever is the shorter.
(DOCX)

S2 Fig Induced cell cycle arrest leads to increased HBV production in a stably transfected cell line. HBV producing HepG2.2.15 cells were exposed to aphidicolin 10μg/ml to induce G/1S arrest or 5nM camptothecin to induce G2/M arrest. A) Supernatant HBsAg 10 days after cell cycle arrest. B) Supernatant HBV DNA 10 days after cell cycle arrest. C-E) HBcAg expression 10 days after cell cycle arrest in C) control D) G1/S and E) G2/M arrest.
(DOCX)

S1 Table Clinical and demographic data for patients with HBV, normal donor liver and liver regeneration (following transplantation). (NR = not recorded, N/A = not applicable, ALT = Alanine aminotransferase, ALP = Alkaline phosphatase, F5–6 = fibrosis stage 5–6)
(DOCX)

S2 Table Antibodies used in immunohistochemistry and immunofluorescence. (DOCX)

## References

[1] American Association for the Study of Liver Diseases Chronic Hepatitis B practice guideline. Available at: http://www.aasld.org/practiceguidelines/Documents/Bookmarked%20Practice%20Guidelines/Chronic_Hep_B_Update_2009%208_24_2009.pdf

[2] Yim HJ, Lok AS. Natural history of chronic hepatitis B virus infection: what we knew in 1981 and what we know in 2005. Hepatology 2006;43: S173–81. 16447285 10.1002/hep.20956

[3] Naoumov NV, Portmann BC, Tedder RS, Ferns B, Eddleston AL, Alexander GJ et al Detection of hepatitis B virus antigens in liver tissue. A relation to viral replication and histology in chronic hepatitis B infection. Gastroenterology 1990;99(4):1248–53 2203664 10.1016/0016-5085(90)90811-e

[4] Wu PC, Lau JY, Lau TK, Lau SK, Lai CL. Relationship between intrahepatic expression of hepatitis B viral antigens and histology in Chinese patients with chronic hepatitis B virus infection. Am J Clin Pathol 1993 12;100(6):648–53. 8249912 10.1093/ajcp/100.6.648

[5] Wiemann SU, Satyanarayana A, Tsahuridu M, Tilmann HL, Zender L, Klempnauer J et al Hepatocyte telomere shortening and senescence are general markers of human liver cirrhosis. FASEB J 2002;16:935–942. 12087054 10.1096/fj.01-0977com

[6] Marshall A, Rushbrook S, Davies SE, Morris LS, Scott IS, Vowler SL et al Relation between hepatocyte G1 arrest, impaired hepatic regeneration, and fibrosis in chronic hepatitis C virus infection. Gastroenterology 2005;128:33–42. 15633121 10.1053/j.gastro.2004.09.076

[7] Aravinthan A, Scarpini C, Tachtatzis P, Verma S, Penrhyn-Lowe S, Harvey R et al Hepatocyte senescence predicts progression in non-alcohol-related fatty liver disease. J Hepatol 2013 3; 58(3): 549–65. 10.1016/j.jhep.2012.10.031 23142622

[8] Aravinthan A, Pietrosi G, Hoare M, Jupp J, Marshall A, Verrill C et al Hepatocyte expression of the senescence marker p21 is linked to fibrosis and an adverse liver-related outcome in alcohol-related liver disease. PLOS One. 2013 9 23;8(9):e72904 10.1371/journal.pone.0072904 24086266 PMC3781134

[9] Knodell RG, Ishak KG, Black WC, Chen TS, Craig R, Kaplowitz N et al Formulation and application of a numerical scoring system for assessing histological activity in asymptomatic chronic active hepatitis. Hepatology 1981; Sep-Oct;1(5):431–5. 7308988 10.1002/hep.1840010511

[10] Verma S, Tachtatzis P, Penrhyn-Lowe S, Scarpini C, Jurk D, Von Zglinicki T et al Sustained telomere length in hepatocytes and cholangiocytes with increasing age in normal liver. Hepatology 2012;56(4):1510–20 10.1002/hep.25787 22504828

[11] Huang YQ, Wang LW, Yan SN, Gong ZJ. Effects of cell cycle on telomerase activity and on hepatitis B virus replication in HepG2 2.2.15 cells. Hepatobiliary Pancreat Dis Int 2004 11;3(4):543–547. 15567742

[12] Bellon M, Nicot C. Regulation of telomerase and telomeres: human tumor viruses take control. J Natl Cancer Inst 2008;100:98–108. 10.1093/jnci/djm269 18182620

[13] Aravinthan A, Verma S, Coleman N, Davies S, Allison M, Alexander G. Vacuolation in hepatocyte nuclei is a marker of senescence. J Clin Pathol 2012;65:557–60 10.1136/jclinpath-2011-200641 22447919

[14] Kim OH, Oh BK, Roncalli M, Park C, Yoon SMA, Yoo JE et al Large liver cell change in hepatitis B virus-related liver cirrhosis. Hepatology 2009 9;50(3):752–762. 10.1002/hep.23072 19585549

[15] Fattovich G. Natural history and prognosis of hepatitis B. Semin Liver Dis 2003; 23: 47–68. 12616450 10.1055/s-2003-37590

[16] Kao JH, Chen DS. HBV genotypes: epidemiology and implications regarding natural history. Curr Hepat Rep 2006; 5: 5–13.

[17] Ferrari C, Penna A, Sansoni P, Giuberti T, Neri TM, Chisari FV et al Selective sensitization of peripheral blood T lymphocytes to hepatitis B core antigen in patients with chronic active hepatitis type B. Clin Exp Immunol 1986; 66: 497–506. 3494552 PMC1542471

[18] Jung MC, Spengler U, Schraut W, Hoffman R, Zachoval R, Eisenburg J et al Hepatitis B virus antigen-specific T-cell activation in patients with acute and chronic hepatitis B. J Hepatol 1991; 13:310–317 1808224 10.1016/0168-8278(91)90074-l

[19] Nayersina R, Fowler P, Guilhot S, Missale G, Cerny A, Schlicht HJ et al HLA A2 restricted cytotoxic T lymphocyte responses to multiple hepatitis B surface antigen epitopes during hepatitis B virus infection. J Immunol 1993; 150: 4659–4671. 7683326

[20] Barnaba V, Franco A, Alberti A, Benvenuto R, Balsano F. Selective killing of hepatitis B envelope antigen-specific B cells by class I restricted, exogenous antigen specific T lymphocytes. Nature 1990;345:258–260. 2110296 10.1038/345258a0

[21] Campisi J, Andersen JK, Kapahi P, Melov S. Cellular senescence: a link between cancer and age-related degenerative disease? Semin Cancer Biol. 2011 12;21(6):354–9 10.1016/j.semcancer.2011.09.001 21925603 PMC3230665

[22] Borel F, Lacroix FB, Margolis RL. Prolonged arrest of mammalian cells at the G1/S boundary results in permanent S phase stasis. J Cell Sci. 2002;115:2829–38. 12082144 10.1242/jcs.115.14.2829

