# Correction: Chronic Hepatitis B Virus Infection: The Relation between Hepatitis B Antigen Expression, Telomere Length, Senescence, Inflammation and Fibrosis

**DOI:** 10.1371/journal.pone.0134315

**Published:** 2015-07-28

**Authors:** Phaedra M. Tachtatzis, Aileen Marshall, Aloysious Aravinthan, Suman Verma, Sue Penrhyn-Lowe, Marianna Mela, Cinzia Scarpini, Susan E. Davies, Nicholas Coleman, Graeme J. M. Alexander

The third author’s name in the author byline is incorrect. The correct name is Aloysious Arvinthan. The article citation remains correct.
