# Supplementary figures and images for: Chronic Hepatitis B Virus Infection: The Relation between Hepatitis B Antigen Expression, Telomere Length, Senescence, Inflammation and Fibrosis
Source: PLoS One. 2015 May 29;10(5):e0127511. doi: 10.1371/journal.pone.0127511 (PMC4449162; doi:10.1371/journal.pone.0127511)

**
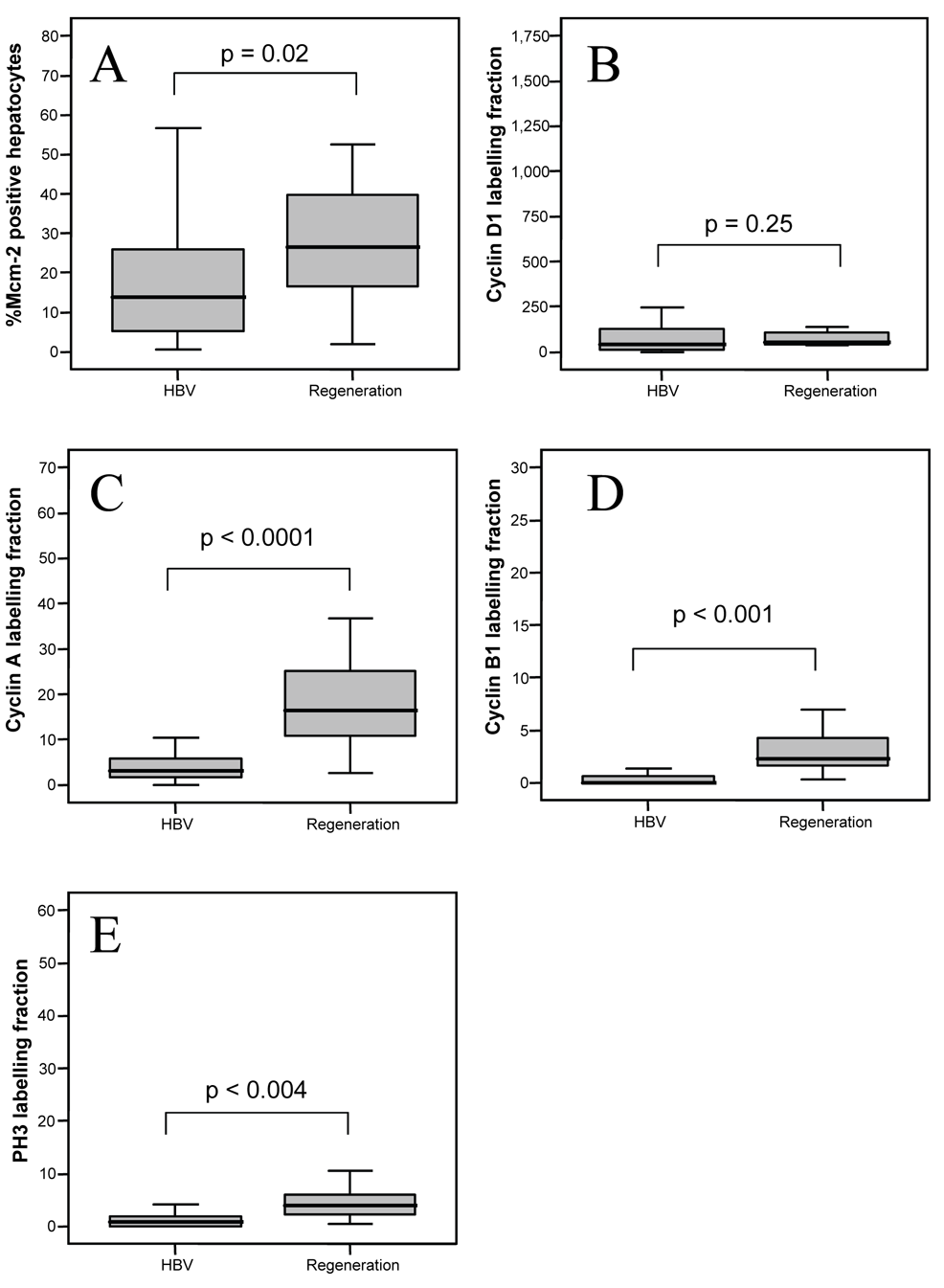
**

**S1 Figure**

Supplement: S1 Fig — (A) % Mcm-2 positive hepatocytes in HBV versus liver regeneration, p = 0.02. (B)–(E) show results expressed as % of Mcm-2 positive hepatocytes for each case. (B) Cyclin D1, p = 0.25 (C) Cyclin A, p < 0.0001 (D) Cyclin B1, p < 0.0001 (E) PH3, p = 0. 0004. The black bar in the middle of the box represents the median, the box stretches between the lower and upper quartiles and the whiskers extend to the range of the data or 1.5 times the box length whichever is the shorter. (DOCX) [file pone.0127511.s001.docx]

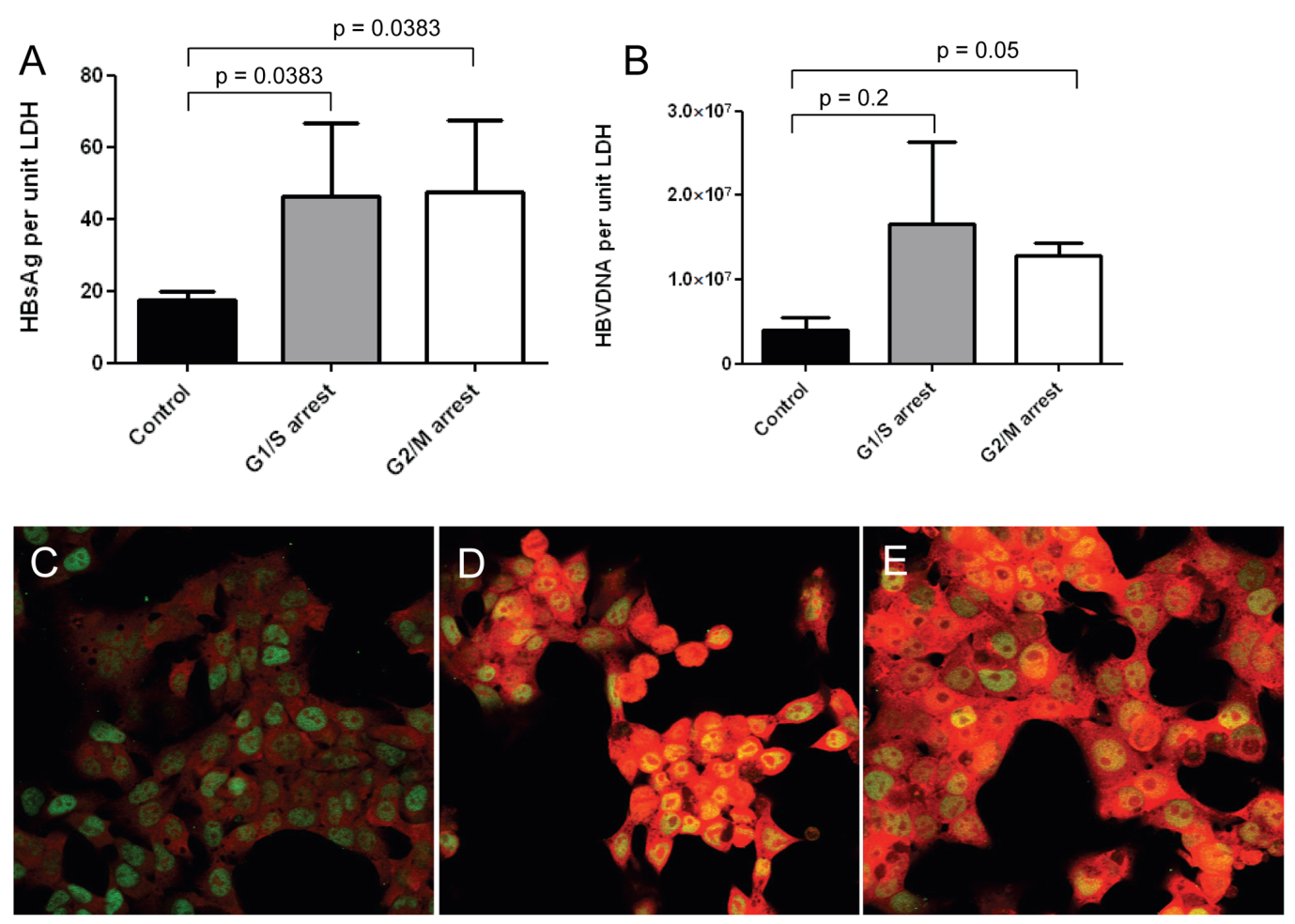


**S2 Figure**

Supplement: S2 Fig — HBV producing HepG2.2.15 cells were exposed to aphidicolin 10μg/ml to induce G/1S arrest or 5nM camptothecin to induce G2/M arrest. A) Supernatant HBsAg 10 days after cell cycle arrest. B) Supernatant HBV DNA 10 days after cell cycle arrest. C-E) HBcAg expression 10 days after cell cycle arrest in C) control D) G1/S and E) G2/M arrest. (DOCX) [file pone.0127511.s002.docx]
