# Supplementary material for: Chronic Hepatitis B Virus Infection: The Relation between Hepatitis B Antigen Expression, Telomere Length, Senescence, Inflammation and Fibrosis
Source: PLoS One. 2015 May 29;10(5):e0127511. doi: 10.1371/journal.pone.0127511 (PMC4449162; doi:10.1371/journal.pone.0127511)
Supplement: S1 Table — (NR = not recorded, N/A = not applicable, ALT = Alanine aminotransferase, ALP = Alkaline phosphatase, F5–6 = fibrosis stage 5–6) (DOCX) [file pone.0127511.s003.docx]

|  | Chronic HBV | Normal donor liver (time zero) | Liver regeneration |
| --- | --- | --- | --- |
| Mean age, years  (range) | 38.9  (18 – 74) | 45  (5 – 77) | 54  (37 – 63) |
| Gender (% male) | 75 | 52 | 40 |
| Cirrhosis (F 5-6) (%) | 32 | 0 | 0 |
| Portal inflammation  (% score 3-4) | 15 | N/A | NR |
| Interface hepatitis  (% score 3-4) | 10 | N/A | NR |
| Median bilirubin  (<21 mg/dL) | 10 | NR | 27 |
| Median ALT  (<50 IU/L) | 75 | NR | 191 |

**S1 Table**
