# Supplementary material for: Chronic Hepatitis B Virus Infection: The Relation between Hepatitis B Antigen Expression, Telomere Length, Senescence, Inflammation and Fibrosis
Source: PLoS One. 2015 May 29;10(5):e0127511. doi: 10.1371/journal.pone.0127511 (PMC4449162; doi:10.1371/journal.pone.0127511)
Supplement: S2 Table — (DOCX) [file pone.0127511.s004.docx]

|  | Antigen | Antibody manufacturer | Antibody type | Antibody concentration | Antigen retrieval buffer |
| --- | --- | --- | --- | --- | --- |
| Cell cycle phase markers (Immunohistochemistry) | MCM-2 - all phases | In-house | Mouse monoclonal | 1 : 8 | 10mM citrate |
|  | Cyclin D1 - G1 phase | Dako | Mouse monoclonal | 1 : 25 | Dako high pH antigen retrieval buffer |
|  | Cyclin A - S phase | Novocastra | Mouse monoclonal | 1 : 50 | 10mM citrate |
|  | Cyclin B1 - G2 | Dako | Mouse monoclonal | 1 : 25 | 10mM citrate |
|  | Phosphorylated histone 3 - mitosis | Upstate Biotechnology | Rabbit polyclonal | 1 : 500 | 10mM citrate |
|  | p21 | Dako | Mouse monoclonal | 1 : 100 | EDTA |
|  | HepB core protein | Dako | Rabbit polyclonal | 1 : 100 | 10mM citrate |
| Hepatocytes (Immunofluorescence) | Hepar 1 | Dako | Mouse monoclonal | 1 : 100 | 10mM citrate |
| Hepatitis B antigens (Immunofluorescence) | Surface antigen | Dako | Mouse monoclonal | 1 : 5 | 10mM citrate |
|  | Core antigen | Abcam | Mouse monoclonal | 1 : 5 | 10mM citrate |
|  | E antigen | Gift: Prof R Tedder (Ref) | Mouse monoclonal | 1 : 5 | 10mM citrate |

**S2 Table**
